# Carbonate Precipitation through Microbial Activities in Natural Environment, and Their Potential in Biotechnology: A Review

**DOI:** 10.3389/fbioe.2016.00004

**Published:** 2016-01-20

**Authors:** Tingting Zhu, Maria Dittrich

**Affiliations:** ^1^Department of Physical and Environmental Sciences, University of Toronto Scarborough, Toronto, ON, Canada

**Keywords:** microbial carbonate precipitation, metabolisms, natural environment, biotechnology, challenges

## Abstract

Calcium carbonate represents a large portion of carbon reservoir and is used commercially for a variety of applications. Microbial carbonate precipitation, a by-product of microbial activities, plays an important metal coprecipitation and cementation role in natural systems. This natural process occurring in various geological settings can be mimicked and used for a number of biotechnologies, such as metal remediation, carbon sequestration, enhanced oil recovery, and construction restoration. In this study, different metabolic activities leading to calcium carbonate precipitation, their native environment, and potential applications and challenges are reviewed.

## Introduction

Carbonates in the form of limestone and dolomite represent an important carbon reservoir, accounting for 41.9% of the total carbon on Earth (Ehrlich, 1996). At the Earth’s surface, a significant portion of the insoluble carbonate is of biogenic origin, involving bacteria, fungi, algae, and metazoa (Gadd, 2010). The involvement of microorganisms in the massive precipitation of calcium carbonates is well illustrated by stromatolites (Grotzinger and Knoll, 1999) and whiting events (Thompson et al., 1997). Microbial carbonate precipitation (MCP) can occur as a by-product of common microbial metabolic activities, including photosynthesis (Dupraz et al., 2004), ureolysis (Fujita et al., 2000), denitrification (Van Paassen et al., 2010), ammonification (Rodriguez-Navarro et al., 2003), sulfate reduction (Braissant et al., 2007), and methane oxidation (Reeburgh, 2007). In addition, cell walls and extracellular polymeric substances (EPS) are reported to serve as templates for carbonate precipitation (Obst et al., 2009).

A stunningly diverse collection of microbial species is nurtured by a variety of environmental conditions. MCP is an ubiquitous process that plays an important role in the cementation of natural systems, such as caves, soils, sediments, aquifers, and open-water areas (Riding, 2000; Banks et al., 2010; Chou et al., 2011). Inspired by its natural capability to coprecipitate metal ions, cement sands/soils/minerals, and sequestrate CO_2_, MCP is proposed to be a promising technology in metal remediation, soil reinforcement, enhanced oil recovery (EOR), and construction restoration (Dejong et al., 2006; Sen, 2008; Bang et al., 2010; Achal et al., 2012).

Although successful technological applications of MCP have been demonstrated in the laboratory, challenges still exist regarding upscaling these processes to the field scale, and managing the treatment of by-products (De Jong et al., 2013). Few studies have attempted to identify the environmental factors that exert the strongest influences on microbial communities, which are most diverse and abundant on Earth (Fierer and Jackson, 2006). Compared with the experimental conditions in the laboratory, those of the fields are much more complex and interfered with each other, hence exerting more inhibitions and triggers on microbial growth and metabolic activities.

A majority of the studies on biotechnology using MCP, especially in metal remediation, microbial enhanced oil recovery (MEOR), and construction restoration, are based on ureolysis (De Muynck et al., 2013; Kumari et al., 2014). However, ureolytic bacteria are not ubiquitous, considering specific environmental conditions. In many applications, their growth or metabolic activities are inhibited, and in other cases, they are not able to survive (Dhami et al., 2013; Phillips et al., 2013; Achal and Mukherjee, 2015). Therefore, alternative microbial communities leading to calcium carbonate precipitation should be suggested and considered for the best option of engineering projects.

This study reviews different microbial activities related to calcium carbonate precipitation, their occurrence in various geological settings, the application fields of MCP biotechnology, and the challenges for the real applications. A relationship between metabolic pathways and environmental conditions is established, its indication for the potential application is presented, challenges related to different engineering projects are identified, and strategies to mitigate those challenges are proposed.

## Microbial Carbonate Precipitation by Different Microbial Activities in Natural Environments

In nature, a variety of microorganisms is known to induce carbonate precipitation by altering solution chemistry through a wide range of physiological activities (Castanier et al., 1999; Riding, 2000; Dejong et al., 2010; Maignien et al., 2011; De Muynck et al., 2013), or by serving as a crystal nucleus (Aloisi et al., 2006). In marine and freshwater systems, photosynthetic microbes are responsible for triggering calcite precipitation (Arp et al., 2001; Dittrich and Obst, 2004; Plée et al., 2010), whereas in a wide variety of environments, consortia with both phototrophs (e.g., cyanobacteria and microalgae) and heterotrophs mediate the calcite precipitation (Power et al., 2011). Microorganisms induce carbonate precipitation through different metabolic pathways, such as photosynthesis, ureolysis, ammonification, denitrification, sulfate reduction, anaerobic sulfide oxidation, and methane oxidation, either increasing pH or dissolved inorganic carbon (DIC) (Table 1). Furthermore, most microbial cells provide nucleation sites for carbonate formation. Inspired by their precipitation potential in nature, several biotechnologies related to MCP have been proposed (Whiffin et al., 2007; Fujita et al., 2008; De Belie and De Muynck, 2009; Bang et al., 2010).

**Table 1:** **Reactions and by-products involved in different metabolic pathways leading to MCP**.

Microbial groups	Metabolism	Reference	Reactions	By-product
Cyanobacteria	Photosynthesis	Baumgartner et al. (2006)	$2{\text{HCO}}_{3}^{-}$ + Ca^2+^ → CH_2_O + CaCO_3_ + O_2_	O_2_
Algae				CH_2_O
Ureolytic bacteria	Ureolysis	Achal and Mukherjee (2015)	CO(NH_2_)_2_ + 2H_2_O + Ca^2+^ + Cell → $2{\text{NH}}_{4}^{+}$ + Cell-CaCO_3_	${\text{NH}}_{4}^{+}$
Nitrate-reducing bacteria	Denitrification	Erşan et al. (2015b)	CH_2_COO^−^ + 2.6H^+^ + $1.6{\text{NO}}_{3}^{-}$ → 2CO_2_ + 0.8N_2_ + 2.8H_2_O	Complete reaction: CO_2_ + N_2_
			Ca^2+^ + CO_2_(aq) + 2OH^−^ → CaCO_3_(s) + H_2_O	Incomplete reaction: NO + N_2_O
Myxobacteria	Ammonification	González-Muñoz et al. (2010)	–	NH_3_
Sulfate reduction bacteria	Sulfate reduction	Baumgartner et al. (2006)	${\text{SO}}_{4}^{2-}$ + 2[CH_2_O] + OH^−^ + Ca^2+^ → CaCO_3_ + CO_2_ + 2H_2_O + HS^−^	CO_2_
				HS^−^
Methanogens	Methane oxidation	Reeburgh (2007)	Anaerobic oxidation: CH_4_ + ${\text{SO}}_{4}^{2-}$ + Ca^2+^ → CaCO_3_ + H_2_S + H_2_O Aerobic oxidation: CH_4_ + 2O_2_ → CO_2_ + 2H_2_O	H_2_S

**Figure 1:**
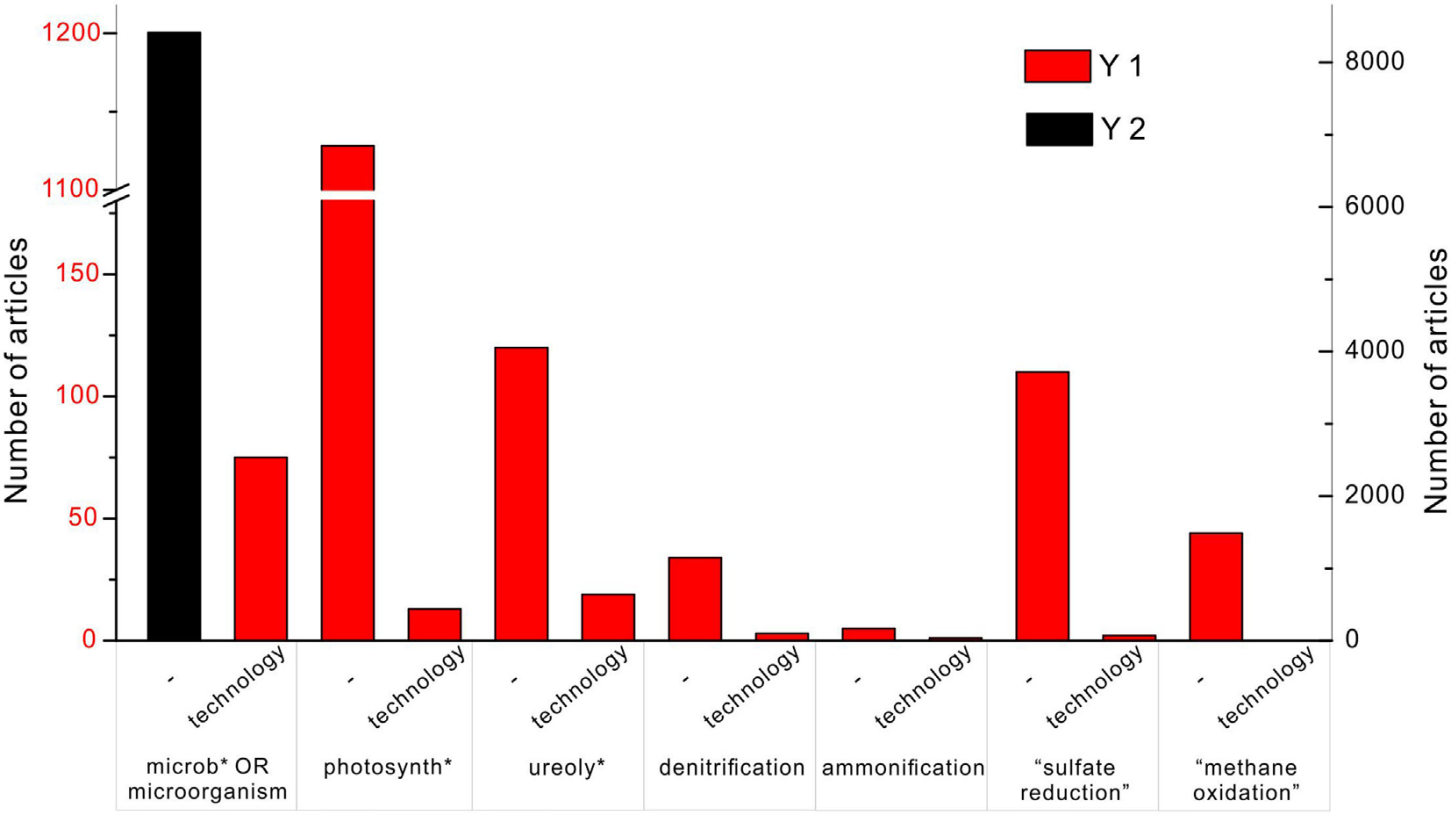
**Number of articles on the topic of “carbonate precipitation” OR “calcite precipitation” OR “calcification” in the database of Web of Science of all years**. The black column represents the search with an additional keyword “microb* OR microorganisms,” and corresponds to the *y*-axis on the right side. The red column indicates the number of publications found with an additional keyword “technology,” and corresponds to the *y*-axis on the left showing 75 articles. Among published work, carbonate precipitation induced by photosynthesis, ureolysis, and sulfate reduction are well studied with 1128, 120, and 110 articles, respectively. Technologies of microbial carbonate precipitation are commonly based on photosynthesis and ureolysis, with 13 and 19 articles, respectively.

The importance of the MCP is reflected in the tremendous amount of publications, and some studies have tested MCP for the real applications. In the database Web of Science, the search topic (“carbonate precipitation” OR “calcite precipitation” OR calcification) AND (microb* OR microorganisms) shows 8412 results (Figure 1). Adding one more keyword – “technology” – to the search, results in 75 articles. Replacing the keyword (microb* OR microorganisms) with photosynth*, ureoly*, denitrification, ammonification, “sulfate reduction,” and “methane oxidation,” the number of the articles is 1128, 120, 34, 5, 110, and 44, respectively. It indicates that the mostly studied calcification processes in the natural environment are through photosynthesis and ureolysis. By adding “technology” to each of them, it results in 13, 19, 3, 1, 2, and 0 articles instead. It shows that ureolysis is the most developed technology among other metabolisms, followed by photosynthesis. Although “sulfate reduction” is widely studied, it is less likely to be taken advantage of in the engineering project.

### Carbonate Precipitation Induced by Microbes

#### Cell Surfaces and Extracellular Polymeric Substances

**Figure 2:**
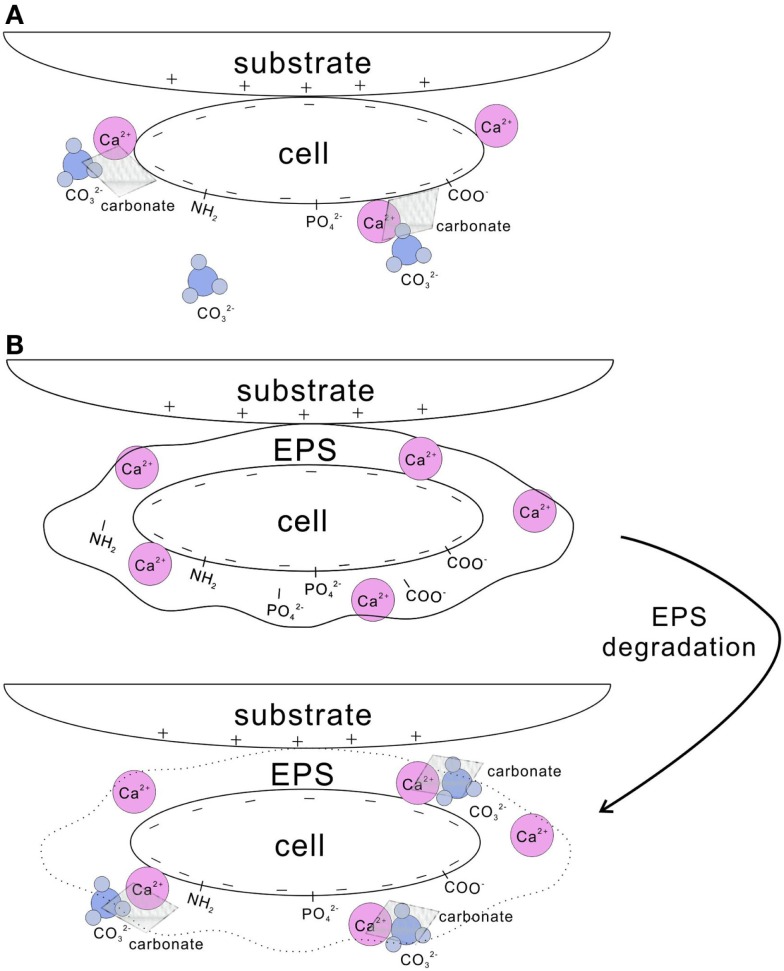
**Nucleation of carbonate crystals on microbial surfaces**. **(A)** Cell wall with negatively charged functional groups, such as carboxyl, phosphate, and amine groups, adsorbs Ca^2+^. Subsequently, carbonates precipitates on the cell surface when carbonate species are available. **(B)** EPS-containing functional groups trap a large amount of Ca^2+^. After EPS is degraded, high concentration of Ca^2+^ is reached locally and results in the precipitation of calcium carbonate in the presence of carbonate species. In addition, cells with negatively charged surface tend to attach to substrates with positive charges.

Cell surfaces have been shown as highly effective nucleation templates for carbonate precipitation (Warren et al., 2001). Cell walls with negatively charged functional groups, such as carboxyl, phosphate, and amine, are able to adsorb metal ions (Dittrich and Sibler, 2005; Fein, 2006) (Figure 2A). For example, the cell wall of *Bacillus subtilis* uptakes a substantial amount of Mg^2+^, Fe^3+^, Cu^2+^, Na^+^, K^+^, Mn^2+^, Zn^2+^, Ca^2+^, Au^3+^, and Ni^2+^ (Beveridge and Murray, 1976; Fein et al., 1997). This increases the concentration of the metal ions in the microenvironment, and when bicarbonate or carbonate is available, the oversaturation in respect to carbonates is easily obtained. In a number of studies, this mechanism was found to be behind calcite nucleation on the cell wall of picocyanobacteria (Warren et al., 2001; Kosamu and Obst, 2009).

Extracellular polymeric substances play an important role in microbial calcification as well (Tourney and Ngwenya, 2014), either inhibiting or promoting carbonate precipitation (Dupraz et al., 2009a). Both photoautotrophic and heterotrophic bacteria can produce EPS, although cyanobacteria are recognized as the most important EPS producers (De Philippis et al., 2001). EPS containing various acidic residues and sugars may trap a large amount of divalent cations, such as Ca^2+^ and Mg^2+^ (Kremer et al., 2008). Metal binders, such as carboxyl, phosphate, amine, and hydroxyl groups, present in EPS (Tourney et al., 2008; Dittrich and Sibler, 2010) (Figure 2B). EPS remove free cations from solution by binding them to those negatively charged groups. Consequently, EPS reduce the saturation in respect of calcium carbonate and inhibit the precipitation of carbonates precipitation (Dupraz et al., 2009a). After EPS are degraded; however, Ca^2+^ may locally reach high concentrations and thus favor precipitation of calcium carbonate (Reid et al., 2000; Dupraz and Visscher, 2005). Carbonates can be precipitated on the non-degraded EPS matrix as well, through a continuous supply of cations combined with a local alkaline condition (Arp et al., 1999). Compared to the abiotically precipitated calcium carbonates, the morphology and mineralogy of those formed with the involvement of EPS vary greatly (Tourney and Ngwenya, 2009). Containing various functional groups, EPS significantly influence bacterial adhesion onto solid substratum surfaces (Figure 2) (Neu, 1996; Tsuneda et al., 2003). In addition, EPS improve the cohesion of sediments by gluing the particles in a similar way EPS support forming microbialites (Tourney and Ngwenya, 2014). Microorganisms adhere to substrates either by generic physical–chemical forces, such as van der Waals and electrostatic forces, or by the specific surface structures of the cell, such as pili, fimbriae, or other appendages (Van Loosdrecht et al., 1989). Determined by surface speciation of EPS, the bacterial and mineral surfaces, hydrophobicity, and electrostatic interactions drive the adsorption of bacteria on mineral surfaces (Yee et al., 2000).

#### Photosynthesis

Photosynthetic microbes, particularly cyanobacteria, are recognized as being responsible for massive carbonate precipitations. It is estimated that cyanobacteria are the principle contributors to the production of carbonate rocks during almost 70% of Earth history (Altermann et al., 2006). Cyanobacterial stromatolite, a laminated calcareous fossil, was found in various environments, such as freshwater, marine, and terrestrial areas (Krumbein and Giele, 1979; Wright, 1989; Goh et al., 2010; Rodriguez-Martinez et al., 2012). Whiting events, which turn the entire water bodies into a milky state (Thompson et al., 1997), also show the high potential of calcification by picocyanobacteria.

**Figure 3:**
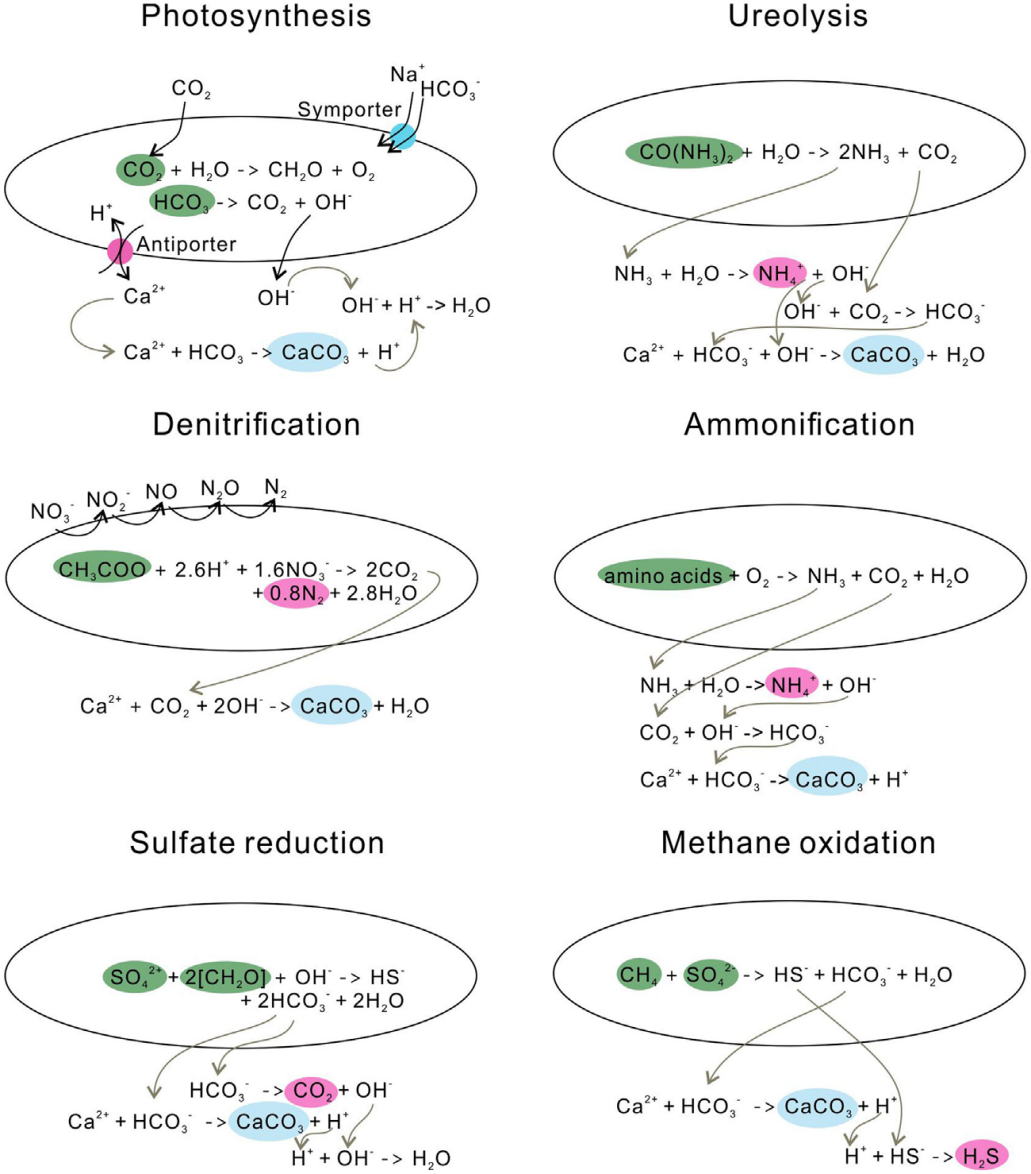
**Microbial carbonate precipitation induced by different metabolisms**.

Photosynthesis leads to calcite precipitation by conducting an ${\text{HCO}}_{3}^{-}$/OH^−^ exchange process across the cell membrane, resulting in an increase of pH in the microenvironment around cells (Miller and Colman, 1980) (Figure 3). Na^+^ and ${\text{HCO}}_{3}^{-}$ are transported into cells through a symporter; CO_2_ enters the cell wall either through this symporter or by diffusion (Espie and Kandasamy, 1992). CO_2_ is synthesized to form organic matter through photosynthesis, and bicarbonate is converted to CO_2_ and OH^−^, the so-called CO_2_ concentration mechanism. The OH^−^ is then released into the solution, increasing the pH (Table 1).

As a result of photosynthetic activity, a higher pH value and the subsequent higher carbonate concentration were measured in illuminated mats than in those measured in dark mats (Ludwig et al., 2005). A mutated-strain modified CO_2_ concentration mechanism resulted in higher ${\text{HCO}}_{3}^{-}$ uptake than wild-type cells, and an increase of the pH of the microenvironment around the cells, increasing the calcification process (Jiang et al., 2013). Interestingly, in some cases, cells are able to store excessive Ca^2+^, precipitate calcium carbonate inside or transport it outside of the cell wall through an antiporter (Waditee et al., 2004).

#### Ureolysis

Similar to the photosynthetic microorganisms, ureolytic bacteria impact the concentration of the DIC and the pH of an environment, yet through urea hydrolysis. For example, *Sporosarcina pasteurii* (Wei et al., 2015) hydrolyze urea to produce ammonia and carbonic acid (De Muynck et al., 2013) (Figure 3). The subsequent hydrolysis of ammonia increases the pH by producing OH^−^, and the dissociation of carbonic acid generates bicarbonates (Knoll, 2003). In this process, alkalinity is increased through urea hydrolysis at a cost of generating the unfavorable by-product ammonia. Therefore, the reaction favors the precipitation of calcium carbonate in the presence of calcium in solution (Table 1).

Urea is a nitrogen source for a variety of microorganisms belonging to different genus or species, including fungi (*Aspergillus* sp., *Coprinus* sp., *Neurospora* sp., *Penicillium* sp., and *Ustilago* sp.), *Bacillus* (*Bacillus lentus*, *B. pasteurii*, *B. sphaericus*, and *B. subtilis*), *Lactobacillus* (*Lactobacillus reuteri*, *L. animalsi*, *L. fermentum*, *Streptococcus salivarius*, *S. mitior*, *S. thermophilus*, and *Staphylococcus epidermis*), *Nitrosomonas* and *Nitrosospira* species, purple sulfur and non-sulfur bacteria (*Rhodobacter capsulatus*), cyanobacteria (*Anabaena cycadae*, *A. cylindrica*, *A. doliolum*, *A. variabilis*, *Anacystis nidulans*, *Spirulina maxima*, *Nostoc calcicola*, *N. muscorum*, and *Brevibacterium ammoniagenes*), and Actinomycetes (*Streptomyces aureofaciens*) (Hasan, 2000). The microbial urease activity is greatly influenced by temperature, pH (with an optimal of 7–8.7 except for acid-urease activity), concentration of urea and the end product ammonia, carbon source, and incubation period (Hasan, 2000).

The genus *Bacillus* is the most common ureolytic bacteria used for biotechnology. They typically are Gram-positive, aerobic, and rod-shaped prokaryotic cells with a size of 1–10 μm (Martin et al., 2012; Wong, 2015). They are particularly interesting for MCP technology due to their capability of producing CO_2_ paralleled by increasing pH in the surrounding environment (Sel et al., 2015; Wei et al., 2015). Both respirations by cells and the decomposition of urea provide a source of CO_2_. For example, *Bacillus diminuta*, one of the most effective carbonatogenic bacterium, was isolated from decayed building stones (Jroundi et al., 2010). Other *Bacillus* species, such as *B. lentus*, habitat in soil, marine, and sediments (Proom and Knight, 1955; Belliveau et al., 1991; Siefert et al., 2000), are widely used in industry to produce alkaline protease (Jørgensen et al., 2000).

#### Denitrification

Under anaerobic conditions, nitrate is used by microorganisms to oxidize organic compounds for energy and cell growth (Martin et al., 2013). A number of bacteria capable of reducing nitrate, the so-called denitrifies, includes *Alcaligenes*, *Bacillus*, *Denitro bacillus*, *Thiobacillus*, *Pseudomonas*, *Spirillum*, *Micrococcus*, and *Achromobacter* (Karatas, 2008). Denitrifiers are typically facultative anaerobes, providing them flexibility in their growth strategy (Karatas, 2008). Due to its highly negative standard Gibbs free energy (ΔG°), denitrification can be expected to dominate where nitrate and organic carbon is present and O_2_ is limited (Dejong et al., 2010).

The denitrification process increases the pH in the surrounding medium by consuming H^+^, and produces CO_2_, which favors carbonate precipitation (Table 1; Figure 3). Till now, only a few studies have investigated the direct link between denitrification and calcium carbonate formation, and observed calcium carbonate crystals precipitate around the cells during denitrification (Erşan et al., 2015b).

The by-product generated during this process is N_2_, which is harmless (Van Paassen et al., 2010). However, toxic nitrite and nitrous oxide might accumulate if any of four different enzymes involving in the denitrification process are inhibited (Van Paassen et al., 2010).

#### Ammonification of Amino Acids

The ammonification of amino acids through microbial metabolisms produces ${\text{CO}}_{3}^{2-}$ and NH_3_ (González-Muñoz et al., 2010) (Figure 3). The subsequent hydrolysis of NH_3_ generates OH^−^ around cells and leads to a high local supersaturation with respect to calcium carbonate, and, consequently, precipitates calcite or vaterite (González-Muñoz et al., 2010).

Some species, which are representers of myxobacteria, e.g., *Myxococcus xanthus* serve as a nucleation template for carbonate precipitation (Chekroun et al., 2004). Their main characteristics, aside from being Gram-negative, aerobic, non-pathogenic, heterotrophic, and rod-shaped, are that they can use amino acids as their sole energy source (Dworkin, 1993; Gerth et al., 2003). These soil bacteria are abundant in almost all environments and play an essential role in the degradation of organic material (Omar et al., 1995).

#### Sulfate Reduction

Sulfate-reducing bacteria (SRB) reduce sulfate to sulfide while oxidizing organic carbon to bicarbonate (Table 1), during which pH and saturation state are increased (Baumgartner et al., 2006) (Figure 3). On the other hand, SRB can increase the local Ca^2+^ concentration by degrading EPS of cyanobacterial mats or excreting Ca^2+^ from cells (Visscher et al., 2000; Plée et al., 2010). With the increase of ${\text{HCO}}_{3}^{-}$ concentration and the release of Ca^2+^, it favors CaCO_3_ precipitation.

In addition, even the metabolically inactive SRB cells, e.g., *D. desulfuricans* strain G20, may lead to calcium carbonate precipitation by providing heterogeneous nucleation sites (Bosak and Newman, 2003). Both their cell surface and EPS impact the calcium carbonate precipitation (Braissant et al., 2007). In many field studies, carbonate precipitation by SRB was observed in microbial mats underneath the surface layers where cyanobacteria are active (Baumgartner et al., 2006).

#### Methane Oxidation

Anaerobic oxidation of methane favors the precipitation of calcium carbonate, whereas aerobic oxidation of methane leads to the dissolution of carbonates by increasing acidity (Reeburgh, 2007) (Table 1). In the anaerobic oxidation, methane is oxidized to bicarbonate, and sulfate is reduced to HS^−^. In the presence of Ca^2+^, calcium carbonate is precipitated, and hydrogen sulfide is generated (Figure 3).

Indicated by methane profiles, radiotracers, and a stable carbon isotope, a large portion of the methane is converted to CO_2_ through anaerobic oxidation in marine sediments (Boetius et al., 2000). In present-day environments, while aerobic methanotrophs have been identified, no organisms consuming methane anaerobically have ever been isolated (Hinrichs et al., 1999).

### Microbial Carbonate Precipitation in Natural Environments

Environmental conditions influence the native bacterial community, which in turn alters the environment through its metabolic activities (Dupraz et al., 2004). In a wide range of modern and ancient geological environments, such as caves, soils, fresh and marine water, and hot springs, the precipitation of carbonate minerals is impacted and governed by microbes (Krumbein et al., 2003). The carbonate products include marine reefs, tidal flats, lacustrine whitings, fluviatile tufas, hot-spring travertines, and cave crusts, as well as subaerial calcretes and other coatings, grains, and matrices within sediment (Riding, 2000).

Aquatic environments, both marine and freshwaters, have been extensively studied in regards to MCP. This is reflected in the amount of publications on the topic: by searching the common keywords of (“carbonate precipitation” OR “calcite precipitation” OR calcification) AND (microb* OR microorganism), and the additional keyword of soil, cave, freshwater, hypersaline, marine, and “hot spring,” it results in 468, 57, 188, 61, 928, and 18 articles, respectively.

**Figure 4:**
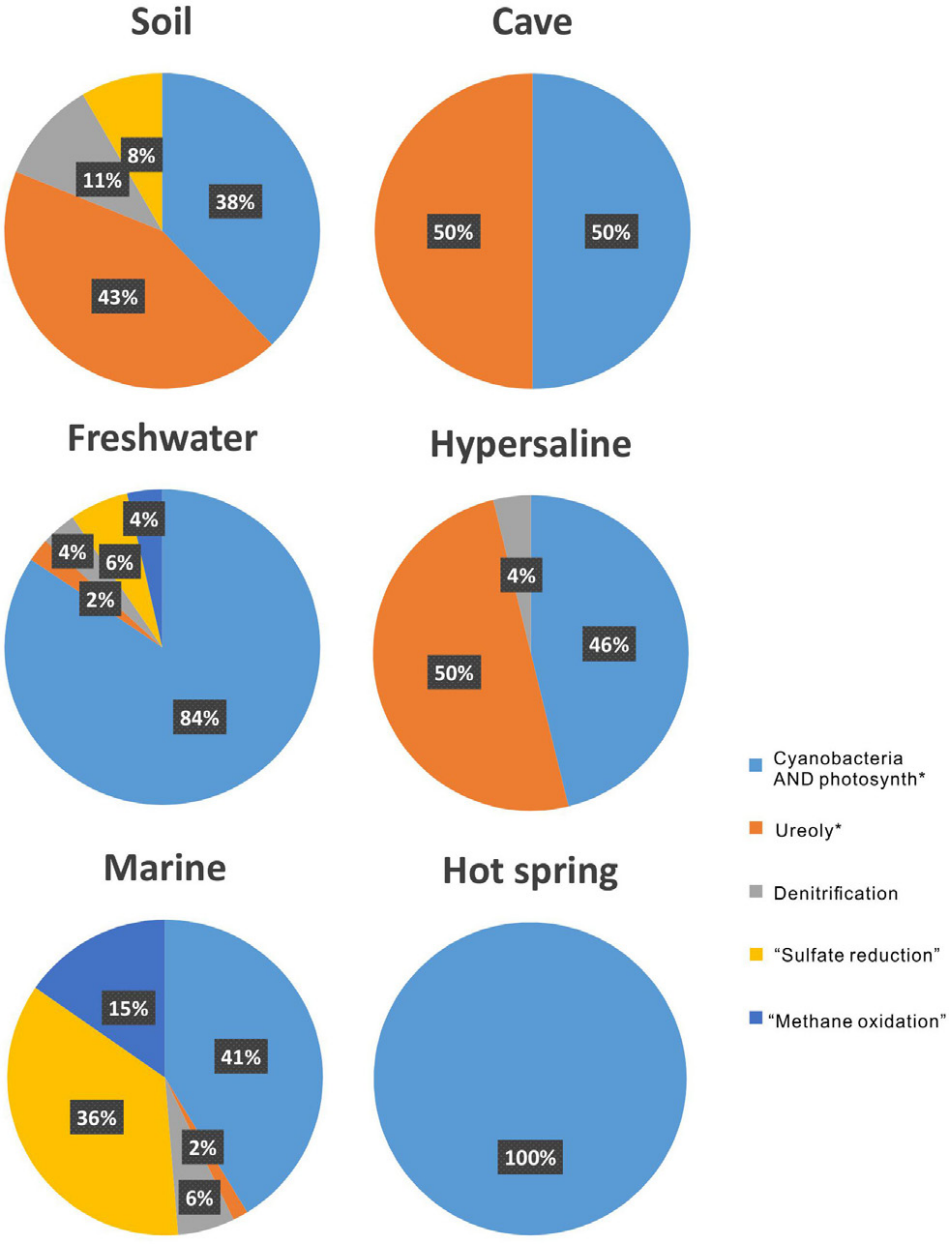
**The proportion of metabolisms that induced microbial carbonate precipitation in each environment**.

In terrestrial systems, the main attention among the metabolic processes has been paid to ureolysis, accounting for 43% among all the metabolisms (Figure 4). “Ureolysis” is followed by “cyanobacteria AND photosynth*,” which accounts for 38%. In the cave and hypersaline environments, both ureolysis and photosynthesis consist of half of the studies on MCP. In freshwater and marine environments, the photosynthesis by cyanobacteria is the most common metabolic process for carbonate precipitation, followed by “sulfate reduction.” In hot-spring environments, only photosynthesis by cyanobacteria is reported.

#### Caves

Caves are characterized by high humidity and relatively low and stable temperature. In many cases, they are oligotrophic environments due to the spatial isolation and low energy supply for any kind of metabolic activities (Gherman et al., 2014). Although calcite formations in caves were believed to be an abiotic process, recent studies suggest that microorganisms may actively participate in the formation of stalactites and stalagmites in caves (Banks et al., 2010). The presence of CO_2_, high calcium concentration, and urea might have contributed to the biomineralization in such habitat (Liu et al., 2010). Notably, in calcium-rich environments, cells have to deal with toxic Ca^2+^ ions as well (Anderson et al., 1992). Indeed, by examining the calcification phenotype of numerous bacterial species and by using a knock-out in the *cha*A calcium antiporter protein, it is suggested that calcification is the Ca^2+^ ions detoxifying strategy taken by cells (Banks et al., 2010).

Photosynthetic microorganisms, such as cyanobacteria, have been implicated in forming stromatolitic carbonate speleothems in the photic zone of carbonate caves (Cañveras et al., 2001; Léveillé et al., 2007). An elevated ^13^C value of calcium carbonate indicates the preferential uptake of ^12^C by photosynthetic microorganisms within the mats (Léveillé et al., 2007).

By introducing urea to caves through the urine of mammals (Johnston et al., 2012), and from infiltration from the surface (Ortiz et al., 2013), it is possible for ureolytic bacteria to grow, sequestrate CO_2_, and precipitate carbonates as well (Okyay and Rodrigues, 2015).

#### Soil

Bacteria are omnipresent in soils at surprisingly high concentrations, almost regardless of saturation, mineralogy, pH, and other environmental factors (De Jong et al., 2013; Sel et al., 2015). Microbial activities can alter the texture of soil and improve its strength by precipitating carbonates and binding soil particles (Sari, 2015). Microbialites have been found in desert soils and tropical forest soils as well (Garcia-Pichel et al., 2003; Cailleau et al., 2004).

Ureolytic bacteria, such as *S. pasteurii*, *Pseudomonas calcis*, *Bacillus* sp., and *Pseudomonas denitrificans*, are widespread in the soil environment, and perform carbonate precipitation (Boquet et al., 1973; Hamdan et al., 2011; Meyer et al., 2011; Chu et al., 2012). Among them, *S. pasteurii* is widely studied due to its highly active urease enzyme, which catalyzes the reaction network toward the precipitation of calcite (Fujita et al., 2000; Hammes et al., 2003).

*Myxococcus*, usually found in soil that is rich in organic matter and decaying material, can precipitate calcium carbonate independently of being metabolically active or killed, though different polymorphs of calcite can be formed (Rodriguez-Navarro et al., 2003; Chekroun et al., 2004).

#### Freshwater Bodies

Depending on the primary production, freshwater bodies can be categorized into oligotrophic, mesotrophic, eutrophic, and hypereutrophic ones. The phenomenon of carbonate precipitation, commonly referred to as whiting, is well known in many eutrophic and mesotrophic lakes, such as Lake Erie and Lake Ontario (Hodell et al., 1998). Moreover, carbonate precipitation can take place even in oligotrophic lakes (Dittrich et al., 2004).

It is suggested that picocyanobacteria are responsible for the carbonate precipitation in these lakes (Dittrich and Obst, 2004). With the impact of the cell surface and photosynthesis, a microenvironment supersaturated with respect to calcite is created (Andrews et al., 1997). In freshwater, the carbonate is usually precipitated on or impregnate with cyanobacterial sheaths or cells (Freytet and Plet, 1996; Freytet and Verrecchia, 1998; Dupraz et al., 2009a).

Calcification of cyanobacterially dominated mats and biofilm can create thick lithified crusts on stream beds and around pebble-sized oncoid nodules (Pedley, 1990; Merz-Preiß and Riding, 1999). In all, calcified cyanobacteria are locally abundant in freshwater streams and lakes (Golubic, 1973). Microbialites, such as travertine, can be found in freshwater lakes (Dupraz et al., 2009a).

#### Hypersaline Lakes

A hypersaline lake contains a significant concentration of sodium chloride or other salts, with saline levels surpassing that of ocean water. Extensive carbonate precipitates associated with microbial mats are quite common in hypersaline lakes (Glunk et al., 2011). For example, carbonate hardgrounds, crusts, and microbialites are abundant in hypersaline lakes, and they can extend from 500 m inland to several tens of meters offshore and into water depths of up to 2 m (Last et al., 2010). Microbialites, from leiolite to thrombolite, have been discovered in hypersaline lakes as well (Dupraz et al., 2004, 2009a).

The mechanism of the MCP in hypersaline lakes is still controversial. Whether it is phototrophs or heterotrophs that play a key role in the calcification is disputable. It is reported that the metabolic reactions of cyanobacteria and SRB promote calcium carbonate precipitation, and those of aerobic heterotrophic bacteria and many sulfide-oxidizing bacteria favor dissolution (Freytet and Verrecchia, 1998; Baumgartner et al., 2009). Both phototrophs and heterotrophs can increase pH through their metabolic activity, and the alteration of the saturation index in mats can represent a combined community effort (Dupraz and Visscher, 2005; Vasconcelos et al., 2014).

According to Ludwig et al. (2005), the increase of Ca^2+^ concentration is mainly due to the EPS degradation by SRB, and the driving factor of carbonate precipitation lies in the increased concentration of ${\text{CO}}_{3}^{2-}$ by photosynthetic bacteria. However, other studies suggest that heterotrophic bacteria, including alkaliphilic and halophilic species, SRB, and ureolytic bacilli, may play a crucial and direct role in carbonate precipitation (López-García et al., 2005).

In addition to influencing the calcium carbonate equilibrium through their metabolism, the microbial community also controls the formation, characteristics, and degradation of EPS, which play an intricate role in the production of calcium carbonate by serving as a template for a crystal growth (Dupraz and Visscher, 2005).

#### Marine Environments

Over geological timescales, the process of CaCO_3_ biomineralization has had a great significance for the fossil record in marine environments (Arp et al., 2001; Riding and Liang, 2005). The forms of carbonate deposits include stromatolites, thrombolites, and carbonated sediment (Riding and Liang, 2005). These formations are a result of calcification by microbial mats and biofilms, commonly dominated by cyanobacteria and/or non-phototrophic bacteria (Arp et al., 2001). The stromatolites in open marine environments are usually laminated and coarse grained (Dupraz et al., 2009a). Both the saturation state of seawater with respect to calcium carbonate (Webb, 2001) and the biomineralization processes (Bosscher and Schlager, 1993) control the marine carbonate precipitation. Microorganisms facilitate CaCO_3_ precipitation by creating a favorable chemical microenvironment for carbonate precipitation by active excretion of Ca^2+^, which can be toxic for cells at seawater concentrations (Banks et al., 2010).

Cyanobacteria (Schoon et al., 2010) and SRB (Visscher et al., 2000) are proposed to promote the calcium carbonate precipitation. The increased alkalinity resulting from the sulfate reduction and Ca^2+^ released from cyanobacterial EPS lead to the CaCO_3_ precipitation (Dupraz et al., 2009a). However, other studies suggested that photosynthetically induced pH and ${\text{CO}}_{3}^{2-}$ increases control calcification, rather than sulfate reduction (Ludwig et al., 2005; Planavsky et al., 2009; Vasconcelos et al., 2014).

#### Hot Springs

Travertine is a common form of limestone deposited in hot springs. Whether it is an abiotic or biotic process that contributes to the formation of travertine is still controversial (Fouke, 2011). Photoautotrophs and heterotrophs are active in hot springs where the temperature is higher than 36.5°C, and in some cases exceed 60°C (Martín and Goldenfeld, 2006; Pedley, 2009), and cyanobacteria have been identified as the main oxygenic photosynthetic microbes (Pentecost, 2003). It is reported that at least three-quarters of carbonate precipitation at 51°C in the Le Zitelle site in Central Italy was microbial mediated (Folk, 1994). With the trace of carbon isotope, it is demonstrated that many dendritic pool precipitates are microbially influenced (Guo et al., 1996). Cyanobacteria, such as *Thermosynechococcus elongatus*, were found to be closely associated with aragonite in the biofilm and microbial mat (Okumura et al., 2013). However, other studies suggest that travertine deposits are produced by a combination of SRB and cyanobacteria metabolic activity (Tekin et al., 2000).

## Potential Biotechnological Applications of MCP

**Figure 5:**
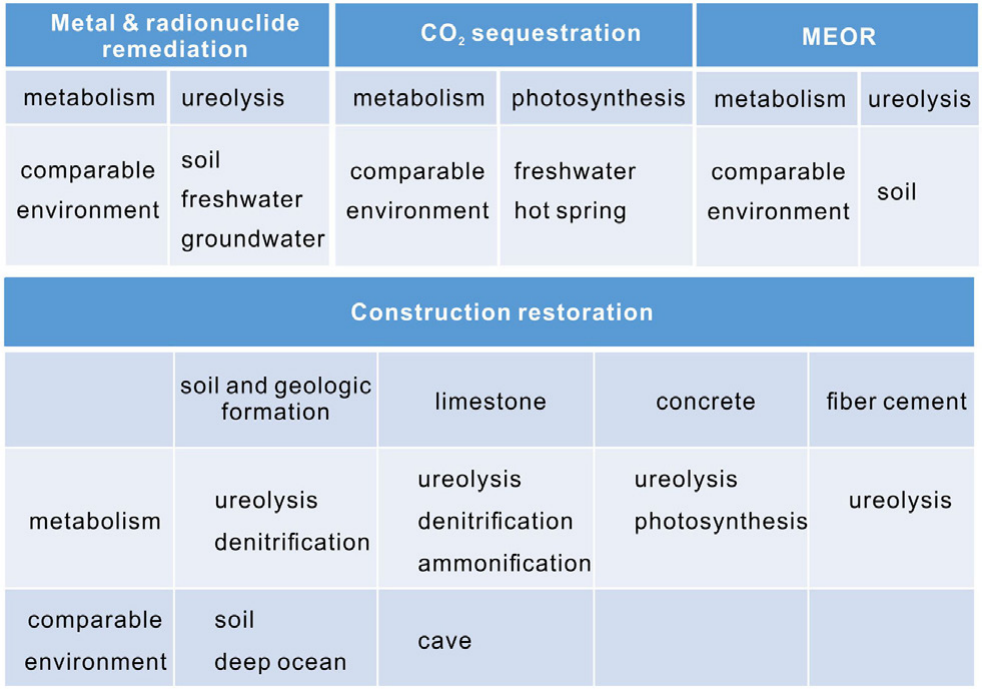
**The application areas of microbial carbonate precipitation, their main metabolisms and comparable environments**.

Calcium carbonate is used commercially for a variety of applications, and yet a wide range of potential uses are under development. For example, the low-cost of calcium carbonate makes it an ideal filler for plastic, rubber, and paper (Sarayu et al., 2014). Recently, microbial precipitated calcium carbonate has shown an advantage by altering the fluorescent property. The calcite crystals formed by thermophilic *Geobacillus thermoglucosidasius* have wide emission wavelengths of 350–600 nm (Yoshida et al., 2010), making them a perfect material as fluorescent particles in stationary ink. Compared with conventional metal-oxide-type phosphors, calcite nucleated by *G. thermoglucosidasius* is more stable and does not require precious rare earth. Instead, they incorporate magnesium, sodium, sulfur, and phosphorus atoms in the carbonate host lattice. It has been suggested that the fluorescence is caused by organic substances, especially humic compounds that is dispersed in the calcite (Lauritzen et al., 1986). The coprecipitation of divalent metals with calcium carbonate can be applied to remove heavy metals and radionuclide (Reeder et al., 1999). The cementitious property makes calcite suitable for CO_2_ sequestration, MEOR, and the restoration of construction materials (Figure 5). Photosynthesis, ureolysis, ammonification, and denitrification induced carbonate precipitation has been applied in these biotechnology (Rodriguez-Navarro et al., 2003; Dick et al., 2006; Ramanan et al., 2010; Erşan et al., 2015b).

### Metal and Radionuclide Remediation

The discharge of heavy metals (e.g., copper, arsenic, and chromium) from mining and metal-processing industries has led to serious contamination in soil and groundwater. Remediation of contaminated sites with a cost-effective and eco-friendly technique is of urgent need. Conventional methods, such as chemical reactions, ion exchange, electrochemical treatment, and membrane technologies are either ineffective or extremely expensive (Ahluwalia and Goyal, 2007). Redox interactions leading to decreased solubility of heavy metals has a risk of remobilization if, in the future, the changes in oxidation–reduction potential occur.

In comparison, CaCO_3_-based coprecipitation offers a stable, redox-insensitive solution (Phillips et al., 2013). Heavy metal ions with an ion radius close to Ca^2+^, such as Sr^2+^, Pb^2+^, Co^2+^, and Zn^2+^, can be incorporated into the calcite crystal by substituting the Ca^2+^ in the lattice, or by entering the interstice or defect of crystal (Reeder et al., 1999; Finch and Allison, 2007).

Ureolysis-involved MCP is a novel remediation approach to capture divalent heavy metals and radionuclides. Although in elevated concentrations, heavy metals may inhibit the growth of ureolytic bacteria (Kurmaç, 2009), the MCP has shown positive results in a highly concentrated heavy metal solution. For example, carbonates induced by *Kocuria flava* removed 97% of copper with an initial Cu concentration of 1000 mg/L (Achal et al., 2011b). MCP induced by *Bacillus cereus* in remediated soil has remarkably decreased the amount of Cr (Kumari et al., 2014), while MCP by *Lysinibacillus sphaericus* removed 99.95% of Cd after incubation of 48 h (Kang et al., 2014). *Sporosarcina ginsengisoli*-induced MCP significantly removed from the aqueous solution (Achal et al., 2012). Furthermore, the efficiency of ureolytic bacteria to bind various types of heavy metal ions is due to their large surface area and availability of anions on the cell surface (Wong, 2015).

Soils and groundwater contaminated with radionuclides, particularly ^137^Cs and ^90^Sr, pose a long-term radiation hazard to human health. Remediation of radionuclide-contaminated land by carbonates has attracted increasing attention. The high stability of carbonates and the potential for coprecipitation of radionuclides is an appealing engineer application (Lauchnor et al., 2013). ^90^Sr, a uranium fission by-product, is readily coprecipitated with CaCO_3_ (Curti, 1999). It is possible for ^90^Sr to decay in sequestrated carbonates due to the short half-life of ^90^Sr (29 years) and the stability of calcite (Lauchnor et al., 2013). MCP induced by ureolytic activity has been examined in detail and has been shown to increase the rate of calcite precipitation and lead to Sr coprecipitation (Fujita et al., 2004; Brookshaw et al., 2012). MCP by *B. pasteurii* captured 95% of total strontium at an initial concentration of 1 mM within 24 h, and the efficiency is much higher than that of UO_2_ and Cu (Warren et al., 2001). By controlling the concentration of carbon source and modifying the injection strategy, even higher Sr coprecipitation efficiency can be achieved (Fujita et al., 2008; Lauchnor et al., 2013).

### CO_2_ Sequestration

The Intergovernmental Panel on Climate Change assessment (Field and Van Aalst, 2014) reported a warming of 0.85°C over the period from 1880 to 2012. Three strategies are proposed to mitigate the climate change (Schrag, 2007): (1) reducing the global energy use, (2) depending on zero-carbon-print energy, and (3) sequestrating CO_2_.

Geological sequestration has a potential risk of upward leakage of CO_2_ through fractures, disturbed rock, or cement lining near injection wells (Cunningham et al., 2009). By contrast, mineral sequestration offers an inexpensive, low-risk, and long-term storage strategy (McCutcheon et al., 2014). Microbial CO_2_ fixation is an emerging and promising technology, and it is considered to be reliable and economically sustainable to mitigate greenhouse gas (GHG) emissions (Rossi et al., 2015). Photosynthetic microbes, such as cyanobacteria and microalgae, play a notable role in carbon sequestration by simultaneously capturing CO_2_, generating power, and producing biodiesel (Wang et al., 2010; Kumar et al., 2011).

Microalgae and cyanobacteria can survive in a wide range of CO_2_ concentrations using CO_2_ concentrating mechanism (CCM) (Badger and Price, 1992). By using *Chlorella* sp. and *Spirulina platensis*, the CO_2_ sequestration efficiency was improved to 46 and 39%, respectively (Ramanan et al., 2010). It is possible to fix CO_2_ at a rate of 33 ton of/C/ha/year in combined mineral and biological storage (McCutcheon et al., 2014). The extensive production of EPS has been demonstrated to be the main factor to accelerate carbonate mineralization (Obst et al., 2009). The biological fixation of CO_2_ can be affected by pH, O_2_ removal, the proper agitation of the reactor, the availability of light, and the appropriate design of bioreactors (Kumar et al., 2011). Compared to vertical tubular and bubble column photobioreactors, airlift-type photobioreactors were found to be the most suitable technique.

No net sequestration of CO_2_(g) was observed through the ureolysis by *S. pasteurii*, indicated by the mole of calcite precipitated being equal to or less than the moles of urea-derived carbonate ions (Mitchell et al., 2010). This is due to the generation of CO_2_ in the process of urea hydrolysis (Figure 3). Nevertheless, MCP through ureolysis provides several engineering advantages, including bioaugmentation of native organisms, utilizing wastewater urea sources, and decreasing the formation porosity.

### Microbial Enhanced Oil Recovery

As an important energy source, crude oil plays a crucial role in economy, politics, and technology. Oil recovery from oil wells is conducted in two phases: (1) about 10–15% of the original oil in place (OOIP) is recovered in the primary phase and (2) about 15% of OOIP is recovered in the secondary phase by water flooding (Suthar et al., 2009). By the end of the conventional oil-recovery methods, almost 65% of oil is left behind in the reservoir. EOR is necessary to improve the overall extraction percentage. However, EOR, which involves thermal or chemical flooding processes, is environmentally hazardous, costly, and leaves undesirable residues that are difficult to dispose (Suthar et al., 2009). In comparison, MEOR offers a cost-effective and eco-friendly strategy. Most commonly, MEOR utilizes microbes to produce biosurfactants, biopolymers, acids, gases, and solvents to reduce interfacial tension between oil-and-water and oil-and-rock interfaces (Sen, 2008). To a lesser extent, MEOR uses bacteria to plug high-permeability zones and lead to a redirection of waterflood, therefore improving the yield of reservoir oil (Dejong et al., 2006).

Inspired by a natural process of microbial carbonate cementing of marine sediments in lagoons and reefs, bacteriogenic mineral plugging to enhance the oil recovery is patented (Ferris and Stehmeier, 1992). The bacterial slurry can be injected into the subsurface environment, predominantly entering highly permeable zones, adhering to the rock/sand surfaces, and precipitating minerals *in situ* to plug porous media (Sen, 2008). EPS producing bacteria selectively block the high-permeability zones, therefore redirect water to flow through the low permeability zones and lead to an increase in oil recovery (Suthar et al., 2009). A variety of *Bacillus* strains are suitable to plug pores by producing EPS and minerals (Sen, 2008). *S. pasteurii* can be used for MEOR, since they naturally occur in the subsurface and do not aggregate, which ensures a high cell surface-to-volume ratio (Dejong et al., 2006). Under subsurface conditions, factors, such as pH, oxygen supply, metabolic status, nutrients, fluid flow, and concentration of microbes and ionic calcium, are critical for the success of the microbial treatment (Fratesi, 2002; Dejong et al., 2006).

### Restoration of Construction Materials

The MCP has shown its merits in improving the mechanical properties of porous materials (Le Metayer-Levrel et al., 1999; Montoya et al., 2013). It not only strengthens underground conditions for constructions, such as soil or geological formation (Whiffin et al., 2007; Harkes et al., 2010; Mahanty et al., 2013), but also restores aboveground structures, such as limestone-based monuments and concrete structures (Tiano et al., 1999; Castanier et al., 2000; Jimenez-Lopez et al., 2007; De Belie and De Muynck, 2009).

#### Soil and Geological Formation Reinforcement

The mechanical properties (cohesion, friction, stiffness, and permeability) are important for soil, which serves as a basis for roads, railways, dikes, dunes, and slopes (Harkes et al., 2010). Stabilization of soil for desired land uses is required, since the mechanical properties of those engineering constructions are typically insufficient. Chemical grouting, one of the soil-strengthening techniques, is costly and requires many injection wells for treating large volumes (Van Paassen et al., 2010). Biogrout, involving MCP *in situ*, provides a sustainable solution.

To cement the porous soil or geological formation by MCP, a large amount of calcium carbonate is needed. However, highly concentrated calcium and inorganic carbon solution in the subsurface would lead to immediate uncontrolled precipitation and would limit injection distance. Furthermore, the simultaneous injection of slightly oversaturated solution of calcium and inorganic carbon would require enormous amounts of liquid volume to induce sufficient precipitation. Therefore, with the injection of a high concentration of dissolved calcium, both inorganic carbon and alkalinity have to be sufficiently produced *in situ* (Van Paassen et al., 2010).

Most of the biogrout studies were based on calcium carbonate precipitation induced by hydrolysis of urea (Harkes et al., 2010). The biomineralization of ureolytic bacteria *S. pasteurii* has significantly increased the soil strength (Dejong et al., 2006). The investigation of biofilm and mineral deposits by *S. pasteurii* in flowing and non-flowing columns shows a potential mitigation of controlling CO_2_ leakage during geological sequestration (Cunningham et al., 2009).

Additionally, other metabolic pathways, such as aerobic oxidation, nitrate reduction, and sulfate reduction, have been investigated for MCP (Van Paassen et al., 2010). Unlike urea hydrolysis generating ammonium chloride as an unfavorable side product, nitrogen gas and CO_2_ produced by the denitrification process are harmless (Van Paassen et al., 2010). With insufficient reaction, however, intermediate compounds, such as nitrite and nitrous oxide, may accumulate (see Denitrification).

Microbial carbonate precipitation was applied in a large-scale soil-improvement project (Whiffin et al., 2007). Techniques, such as the fixation and distribution of bacteria, the balancing of the flow and reaction rate, and the controlling of the injection rate, have been developed to improve cementation uniformity (Whiffin et al., 2007; Harkes et al., 2010; Martinez et al., 2013). The engineering properties of soils, such as permeability, stiffness, shear strength, and volumetric response, have been greatly improved (Dejong et al., 2010; Martinez et al., 2013). Meanwhile, MCP for soil reinforcement reduces cost and the impact on the environment (Dejong et al., 2010).

#### Limestone Restoration

Limestone monuments and buildings are subject to deterioration due to weathering. In order to protect historical objects, many conservation treatments have been introduced to consolidate limestone before irreversible damage occurs. Organic and inorganic materials, such as epoxy resins and Ba(OH)_2_ solutions, treated on deteriorated stones are either adverse to the environment or provide insufficient consolidation (Rodriguez-Navarro et al., 2003). Recently, MCP has been proposed to treat decayed monuments, statues, and buildings.

Ureolytic-driven calcium carbonate precipitation has been applied in limestone restoration. *B. subtillis* was discovered to produce rhomboedral calcite crystals and decrease the stone porosity (Tiano et al., 1999). A comparison of *B. sphaericus* and *B. lentus* demonstrated that *B. sphaericus* strains with a very negative ξ-potential and a high initial urea degradation are most suitable for coherent calcite production on deteriorated limestone (Dick et al., 2006). In 1993, the first application on the tower of the Saint Médard Church exhibited a substantial reduction of water absorption (Le Metayer-Levrel et al., 1999). Another application on the church of Santa Maria in Italy stated that the MCP treatment did not influence the esthetic appearance (Tiano and Cantisani, 2005).

Microbial carbonate precipitation induced by ammonification of amino acids has been applied on decayed limestone as well. *B. cereus*, isolated from soil, was one of the pioneers to be used in preserving a historic property (Castanier et al., 2000). *M. xanthus* is capable of producing a coherent carbonate cement strongly attached to limestone (Rodriguez-Navarro et al., 2003). The organic–inorganic composites make new crystals more stress resistant than the calcite grains of the original stone. Furthermore, the mineralogy and morphology of the precipitates can be manipulated by changing the composition of culture medium.

#### Concrete Restoration

Concrete is the most widely used construction material in the world (Ai¨Tcin, 2000), and its global production is expected to increase until 2050 (Müller and Harnisch, 2008). Even though the durability, cost-effectiveness, high compressive strength, design flexibility, and fire resistance make concrete extraordinarily popular, cracks in concrete are quite common due to freeze–thaw conditions and de-icing chemicals. There are a large number of products available commercially for repairing cracks in concrete, varying from water repellent and stone consolidation to organic treatments and inorganic consolidation. However, these conventional treatments have a number of disadvantages, such as (1) different thermal expansion coefficient of the treated layers, (2) degradation over time, (3) the need for constant maintenance, and (4) the contribution to environmental pollution (De Muynck et al., 2008a).

Recently, researchers proposed the use of MCP in concrete constructions although it is challenging due to the extremely high pH of concrete fluid (Bang et al., 2001). Positive results, such as the improvement of compressive strength, the enhancement of durability, and a decrease of water absorption and permeability, were observed in laboratory studies (Ramakrishnan et al., 1998; De Muynck et al., 2008b; De Belie and De Muynck, 2009). A freeze–thaw cycle experiment demonstrates that the MCP treatment greatly enhances the durability of concrete beams (Bang et al., 2010). A decrease of water absorption rate was observed with the MCP treatment, which improves the resistance of cementations materials toward degradation processes (De Muynck et al., 2008b; Achal et al., 2011a). Compressive strength, one of the most important parameters evaluating the quality of concrete, was significantly increased by the MCP process (Ramachandran et al., 2001; Ramakrishnan, 2007; Bang et al., 2010; Wang et al., 2012). An autonomous repair concrete was developed by casting concrete slabs with *Bacillus* spores and calcium lactate nutrients that are embedded into clay pellets separately (Jonkers and Schlangen, 2009). When cracks form in the concrete, water will enter and open up the pellets, causing the bacteria to germinate and produce calcite (Jonkers and Schlangen, 2009). Since it is a relatively new technology and the concrete life span is around 30 years, the effectiveness of the real application is still under evaluation.

A life cycle-assessment shows that the overall environmental impact of bioconcrete is half that of concrete (Gonsalves, 2011). Bioconcrete excels concrete by having a lower contribution of carcinogens (one-thirtieth), ecotoxicity (one-tenth), climate change (half), and fossil fuels (six-sevenths). The main deficiencies of the ureolytic bioconcrete are the generation of ammonia and the pathogens that are associated with the activity of ureolytic bacteria (Mobley and Hausinger, 1989; Benini et al., 1999). To lower the environmental impact, autophototrophic cyanobacteria were proposed and investigated. They showed a potential to serve as a green technology in concrete restoration (Zhu et al., 2015). It is the first study that shows a thick calcite/cell-aggregate layer precipitated by cyanobacteria on the concrete surface, and decreased water absorption.

#### Fiber Cement

Fiber cement tiles are a fire-resistant and low-cost material usually used for roofing. However, the colonization of biofilm on its surface would reduce its reflectivity (Shirakawa et al., 2014). Recently, carbonate precipitation by ureolytic bacteria has been tested on fiber cement to improve its reflectivity (Shirakawa et al., 2015). After exposure to the environment for 22 months, fiber cement treated with *B. sphaericus* was not degraded by fungi or phototrophs. Therefore, it is a potential treatment to avoid the fouling-associated darkening of fiber cement tiles.

## Challenges in MCP Biotechnology

**Figure 6:**
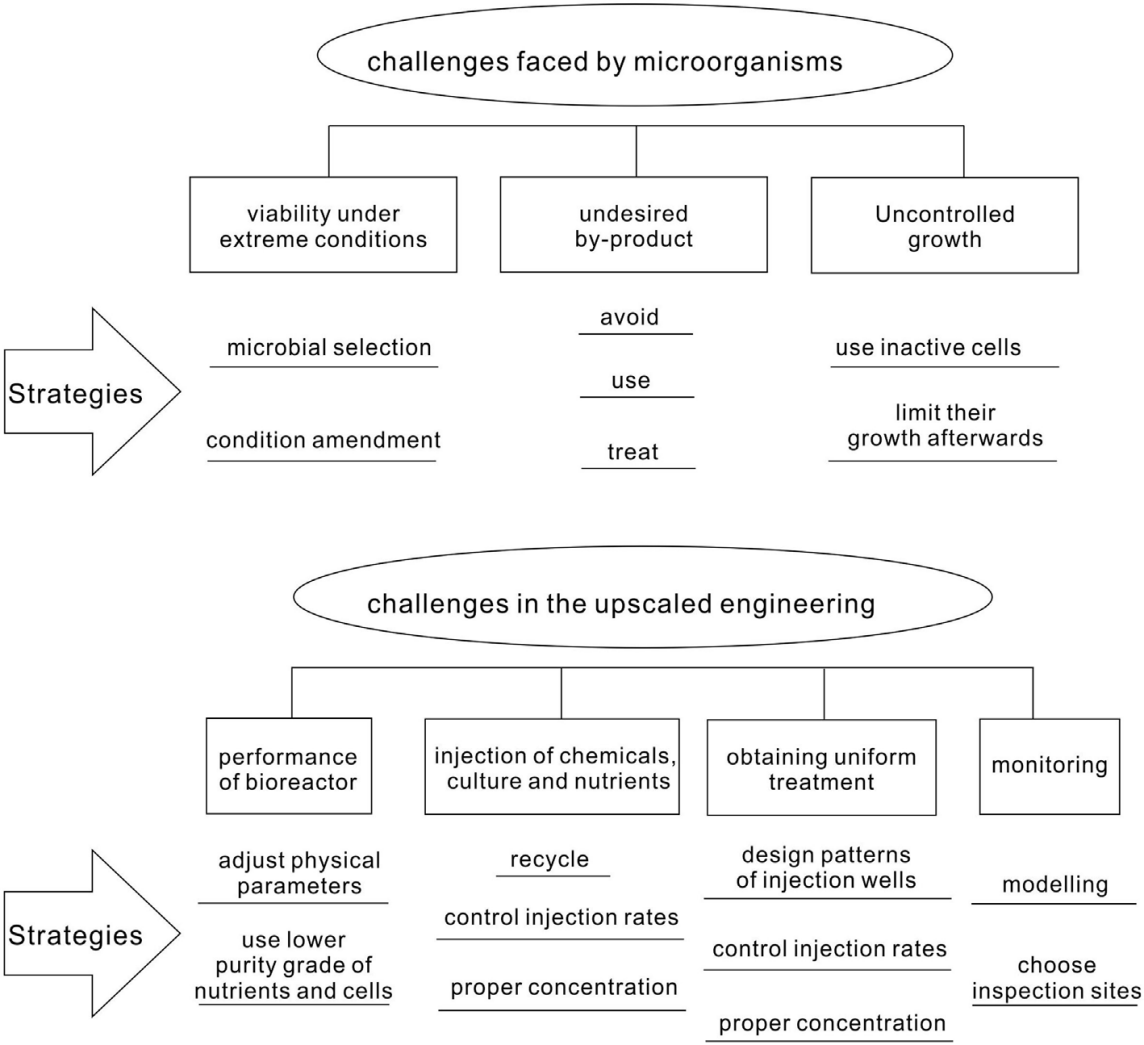
**Challenges faced in the real application of microbial carbonate precipitation**.

Even though MCP has been successfully demonstrated in the laboratory for many cases, its real application in the field is facing numerous challenges (Figure 6). Since the conditions in the field cannot be controlled as in the laboratory, microorganisms are likely to suffer from harsh and changing environments. In addition, the generation of undesired by-products and uncontrolled growth has to be avoided. The upscaling of the MCP from the laboratory condition to the field environment might confront technical difficulties in injecting nutrients, chemicals, and cultures; obtaining homogeneous treatment; and monitoring the remediation process.

### Limitations of Microorganisms

#### Extreme Conditions

In the practical application, microorganisms are often exposed to harsh conditions, such as desiccation, oxygen and nutrient deficiency, high pH, pressure, temperature, and salt concentration.

Desiccation occurs in the MCP application of concrete restoration. When concrete is exposed to ambient air, the moisture content of the concrete structure decreases due to the moisture diffusion (Kim and Lee, 1998). An additional decrease of the moisture content can be caused by the hydration of cement at early ages (Persson, 1997). Besides this restriction for microbial life, the harsh environment of concrete with extremely high pH can be handled by only a few bacteria. These factors lead to the limitation of bacterial strains for concrete restoration. The most widely used bacteria, *S. pasteurii*, is a moderate alkaliphile with a pH optimum for growth of 9.2 (Mobley et al., 1995).

Aerobic microorganisms, such as ureolytic bacteria and myxobacteria, are used in numerous laboratory studies (Rodriguez-Navarro et al., 2003; De Muynck et al., 2010). While their application in limestone and concrete restoration, metal remediation, and soil reinforcement in shallow subsurface are promising, their long-term use under anaerobic conditions, such as deeper parts of geological formation, groundwater, and oil reservoir, are questionable (De Jong et al., 2013). There are still many open questions, e.g., Can they survive until the objective is reached? Even if their growth is not inhibited under this unfavorable condition, are they metabolically active?

Most of the studies conducted in the laboratory conditions were performed under nutrient-rich conditions (Lauchnor et al., 2013), whereas in practical applications, such as soil reinforcement, metal remediation in industrial waste streams, and crack repair in concrete, these conditions are quite rare. It is not clear how cells react to nutrient-deficient environments and whether they continue the carbonate precipitation.

In the case of carbon capture sequestration (CCS), the pressure can be very high. The injection and storage of supercritical CO_2_ into underground geological formation require a minimum pressure at 8 MPa (Martin et al., 2013). Most of the bacteria used in the MCP are cultured under ambient pressure (McCutcheon et al., 2014; Okyay and Rodrigues, 2015). Even though several studies have been conducted at a relatively high pressure at 7.5 MPa (Cunningham et al., 2013; Mitchell et al., 2013), it is still below the minimum requirement for the geological storage of CO_2_. In the experiment, it took 29 days for the system to gradually pressurize to 7.5 MPa (Mitchell et al., 2013), which is not always realistic in the application. It is reported that high pressures significantly inhibit DNA replication and protein synthesis, therefore suppressing the metabolic functions and growth of mesophilic organisms, including *S. pasteurii* (Abe et al., 1999). There is an urgent need to study the impact of high pressure on the MCP.

Other constraints for the broad application are high salinity, elevated temperature, and diminished light intensity. In deep saline aquifers where carbon capture and storage take place, the salt concentration can be as high as 251 g/L (Liu et al., 2012), whereas the salt concentration for growth medium is typically 1000 times lower, between 2.12 and 35 g/L (Ramachandran et al., 2001; Dick et al., 2006; Zhu et al., 2015). The aquifer with a salinity of two orders higher than that in the optimal growth condition of bacteria can be very challenging for microorganisms. The challenges faced by MEOR are the limits on the subsurface environment in which temperature reaches up to 100°C, salinity extends to 9 wt.%, pH ranges between 3 and 9, and pressures goes up to 7000 psi (Ferris and Stehmeier, 1992). Anaerobic and spore-forming microorganisms are preferred under such harsh condition, including genera of the Bacillacae family, such as *Bacillus*, *Sporolactobacillus*, *Clostridium*, *Desulfotomaculum*, and *Sporosarcina* (Ferris et al., 1997). In the pulp and paper industries, the temperature of wastewater can be as high as 60°C (Habets et al., 2000). In the subsurface CO_2_ storage site, the temperature is expected to elevate along the depth (Phillips et al., 2012). For example, during the direct sequestration of CO_2_ in the form of flue gas emitted from power plants, the temperature is around 120°C (Kumar et al., 2011); however, the bacteria used in most of the studies are cultured at a temperature between 20 and 37°C (Warren et al., 2001; Qian et al., 2010). Whether they can survive or maintain their microbial activities is questionable. In the industrial carbon sequestration, where phototrophs are involved, light intensity is essential for MCP (Kumar et al., 2011). While low light intensity limits the biomass productivity, high light intensity causes photo-inhibition of phototrophic microorganisms (Rubio et al., 2003). However, a uniform distribution of light is less likely in most of the cases.

#### By-products and Uncontrolled Growth

Although the MCP excels their comparable “abiotic” application with respect to environmental concerns, it still has a lot of limitations. Toxic ammonium is generated as a by-product during ureolysis (Table 2) and may pose environmental and health risks (De Muynck et al., 2013). To treat the ammonium in groundwater, a higher cost is required.

**Table 2:** **Bacterial species, their metabolic pathways, and possible challenges faced by them in different industrial applications**.

Application	Challenge for bacteria	Bacteria	Metabolic pathway	Reference
Filler for rubber, plastic, and ink		*Geobacillus thermoglucosidasius*	–	Yoshida et al. (2010)
Metal and radionuclide remediation	Toxicity of highly concentrated heavy metal	*Kocuria flava*	Ureolysis	Achal et al. (2011b)
		*Lysinibacillus sphaericus*	Ureolysis	Kang et al. (2014)
		*Sporosarcina ginsengisoli*	Ureolysis	Achal et al. (2012)
		*Bacillus cereus*	Ureolysis	Kumari et al. (2014)
	Radioactivity	*Sporosarcina pasteurii*	Ureolysis	Warren et al. (2001) and Lauchnor et al. (2013)
CO_2_ sequestration	High pressure of CO_2_	*Spirulina platensis*	Photosynthesis	Ramanan et al. (2010) and Kumar et al. (2011)
		*Chlorella vulgaris*	Photosynthesis	Ramanan et al. (2010) and Wang et al. (2010)
		Microbial mat including cyanobacteria and diatoms	Photosynthesis	McCutcheon et al. (2014)
MEOR		*Sporosarcina pasteurii*	Ureolysis	Dejong et al. (2006)
Soil and geological formation reinforcement	Oxic to anoxic condition	*Castellaniella denitrificans*	Denitrification	Van Paassen et al. (2010)
		*Sporosarcina pasteurii*	Ureolysis	Dejong et al. (2006), Whiffin et al. (2007), Harkes et al. (2010), Chu et al. (2012), and Martinez et al. (2013)
		*Halomonas halodenitrificans*	Denitrification	Martin et al. (2013)
	Unique high pressure	*Shewanella frigidimarina*	–	Mitchell et al. (2009)
Limestone restoration		*Myxococcus xanthus*	Ammonification	Rodriguez-Navarro et al. (2003), Chekroun et al. (2004), and González-Muñoz et al. (2010)
		*Bacillus cereus*	Ureolysis/denitrification	Castanier et al. (2000)
		*Sporosarcina pasteurii*	Ureolysis	De Muynck et al. (2013)
		*Bacillus subtilis*	Ureolysis	Tiano et al. (1999)
		*Bacillus sphaericus*	Ureolysis	Dick et al. (2006)
		*Pseudomonas putida*	Ureolysis	May (2005)
		*Bacillus lentus*	Ureolysis	Dick et al. (2006)
		*Micrococcus* sp.	–	Tiano et al. (1999)
		*Pseudomonas*	–	Zamarreno et al. (2009)
		*Acinetobacter*	–	Zamarreno et al. (2009)
Concrete restoration	High pH, desiccation, nutrient deficiency	*Sporosarcina pasteurii*	Ureolysis	Kim et al. (2009) and Bang et al. (2010)
		*Bacillus sphaericus*	Ureolysis	De Belie and De Muynck (2009)
		*Bacillus pseudofirmus*	Ureolysis	Jonkers et al. (2010)
		*Bacillus cohnii*	Ureolysis	Jonkers et al. (2010)
		*Synechococcus*	Photosynthesis	Zhu et al. (2015)

If incomplete reaction occurs during denitrification, a GHG, such as nitrous oxide, will be produced (Van Paassen et al., 2010). With an atmospheric lifetime of 114 years, N_2_O is a powerful GHG that has a 300-fold greater potential for global warming effects compared with that of CO_2_ (Alley et al., 2007; Change, 2007).

The involvement of SRB leads to a release of highly toxic and flammable gas H_2_S, which induces various environmental risks and health symptoms (Kfir et al., 2015). Nausea, vomiting, keratoconjunctivitis, and corneal ulceration occur at a concentration of 50–100 ppm of H_2_S. Olfactory nerve endings become rapidly fatigued when exposed to 100–150 ppm. Rhinitis, bronchitis, and pulmonary edema are caused at a concentration of 200–300 ppm. Cardiopulmonary arrest appears when the concentration is higher than 700 ppm. Unconsciousness and death are serious outcomes at the concentration above 1000 ppm.

Another concern with the MCP biotechnology is the uncontrolled growth of microorganisms. For example, *Bacillus* bacteria may form endospores that can lead to uncontrolled growth (Dei and Chelazzi, 2013). On the other hand, nutrients introduced during the process or organic matters produced by phototrophs can support the growth of environmental microbes, such as airborne fungi (Perito et al., 2014).

### Challenges in the Upscaled Engineering

Successful field implementation of remediation techniques requires upscaling of the systems at the laboratory scale to the field or geological scale. Biomass needs to be produced in an enormous amount, a gigantic bioreactor has to be built in order to consume a large amount of CO_2_, and designs of wells are necessary to inject chemicals/nutrients/cultures into complicated systems. Due to the site-specific conditions and heterogeneous distribution in the field, uniform treatment is harder to be obtained.

Unlike experimental conditions where most parameters can be controlled, more variables in field studies need to be monitored during the application. These variables include microbial consortia or their succession, solution chemistry, and available electron donors. However, to install or design the monitoring system is a tough task not only because of the geological accessibility but also due to the heterogeneous distributions.

#### Upscaling Bioreactors

Laboratory experiments are typically carried out in a 2- to 5-L chemostat, whereas the size of the bioreactor for the field application is several orders of laboratory scale. Therefore, a large volume of nutrients and cultures is required in the industrial application of MCP. Nevertheless, challenges exist not only in producing and transporting the bulky medium and bacterial solution but also in the technical difficulties related to bioreactors. In the treatment of groundwater, possible precipitation can occur in the tubing outside of the reactor or it can be trapped on the filters, resulting in clogging the system (Lauchnor et al., 2013). The case of the waste water treatment shows that the functionality of bioreactors is not well understood, thus the performance of bioreactors is difficult to predict and control (Herrero and Stuckey, 2014). An evenly and efficiently distributed light source is hard to obtain in the photobioreactor for CO_2_ sequestration (Kumar et al., 2011).

The economic concern is another limitation for direct transfer of the laboratory experiments to the field application. For fundamental research in laboratory, analytical chemicals, the highest grade of nutrients, and axenic culture are necessary (Dhami et al., 2013; Silva et al., 2015). However, the use of these expensive materials in the industrial application will result in an extremely high cost.

#### Injection of Chemicals, Nutrients, and Cultures

To ensure the beneficial microbial activities, sufficient nutrients and chemicals need to be supplied. However, the nutrients and chemicals can quickly be depleted due to (1) the rates of flowing through being too fast for reactions to occur, (2) nutrients not being supplied in a sufficient amount, and/or (3) nutrients being used up over time (Dejong et al., 2014).

The depletion rate of nutrients, chemicals, and cultures is a key problem for the calculation of the initial injection amount, and the following amendment injection. Even if nutrients and cultures are supplied in an optimal amount, a restricted transportation can occur due to the extensive precipitation and plugging near the injection well (Fujita et al., 2008). On the other hand, a large amount of incomplete usage of chemicals has been observed after the first flow-through of the column in the laboratory scale. To make it more sustainable, these chemicals can be recycled through the columns after the first injection. However, recirculating the chemicals and nutrients in the field application is confronting technical difficulties. Due to biomineralization, bacteria can die-off or be covered by carbonates overtime, thus becoming inactivated (Cuthbert et al., 2012). Therefore, to seal large voids, such as porous media or rock fractures, additional injections of bacteria are required.

#### Obtaining Uniform Treatment

The precipitation of carbonates is highly impacted by the solution chemistry and microbial activities. Whether it is in groundwater, oil reservoir, soil and geological formation, spatial heterogeneity is expected regarding to solution chemistry, transport, pore distribution, and biodiversity (Dejong et al., 2010). The availability of chemicals, nutrients, and microbes depends upon the fluid movement and transport. Therefore, it is quite challenging to obtain a uniform treatment in the real application.

In the case of groundwater, the injected solution is diluted through the pathway from the injection site to downstream. In the case of soil and geological formation, rapid precipitation occurs around the injection site, plugging the adjacent pores and resulting in blocking the solution to further transport. A field test to improve the geotechnical properties of soil via MCP showed a non-uniform cementation distribution at the initial stage of injection (Dejong et al., 2014).

#### Monitoring

To ensure the high performance and efficiency of the MCP in the field application, parameters such as solution chemistry, microbial activities, and, especially, carbonate production should be monitored. Ion concentrations of the fluid extracted from the bioreactor or column need to be measured to calculate the concentration of calcium and carbonate species; microbial densities are required to be counted to ensure the effectiveness of microbial activities; and solid samples need to be analyzed to calculate the amount of carbonate precipitated as well as their ratio to the adjacent particles. In contrast to the laboratory experiments, parameters, including temperature, pressure, solution chemistry, and microbial communities, vary greatly in the field application. Located in the subsurface, sampling sites are not easily approachable. Therefore, designing monitoring wells and systems are of great challenge.

## Strategies to Overcome the Limitation in MCP-Based Bioremediation Techniques

### Regarding Microbial Cells Limitation

#### Mitigating Extreme Conditions

Here, we are going to discuss the strategies and methods for overcoming the challenges of bioremediation techniques (Figure 6), which were listed in Section “Challenges in MCP Biotechnology.” Bacteria found in natural environment, of which the condition is comparable to that of the field application, can be considered as candidates for MCP. Alternatively, conditions of the field site can be amended to favor the growth of microorganisms being used in the application.

One strategy to mitigate the drying problem is to adopt desiccation-resistant bacteria, which typically form spores (Jonkers and Schlangen, 2009). Another solution is to simultaneously apply protective materials, such as polyurethane and silica gel to immobilize cells (Bang et al., 2001; Kim et al., 2009). Similar materials have been applied to protect bacteria from high pH. In other studies, Cyclic EnRiched Ureolytic Powder and Activated Compact Denitrifying Core are used (Erşan et al., 2015a).

To ensure the viability of aerobic bacteria under anaerobic conditions, air can be injected into the remediation site. However, it is a costly and complicated process. Alternatively, anaerobic denitrifiers offer advantages over ureolytic bacteria by taking nitrate as an oxidant (Martin et al., 2013). Anoxygenic photosynthetic microorganisms, such as *Rhodopseudomonas palustris*, that stimulate the precipitation of calcite can be a good candidate to solve this problem as well (Bosak et al., 2007).

Among the few researchers who have conducted the experiments in low nutrient environment, Erşan et al. (2015b) demonstrated that denitrification strains (*Pseudomonas aeruginosa* and *Diaphorobacter nitroreducens*) were able to precipitate carbonate; in another study, an autotrophic strain of *Synechococcus* has been showed to promote calcification on the concrete surface (Zhu et al., 2015). In addition, a variety of picocyanobacteria demonstrated to stimulate carbonate precipitation in oligotrophic environments (Dittrich et al., 2004; Liu et al., 2010).

Pressure-resistant bacteria are preferred to repair the fractures in the capping rocks. One study using the pressure close to the actual situation of CCS has been conducted so far (Martin et al., 2013). It found that *Halomonas halodenitrificans* can grow under a pressure of 20 MPa, and it is suitable for such application (Martin et al., 2013). Compared with the ambient pressure, the rate of calcium removal by *H. halodenitrificans* was not reduced at 8 MPa. The biofilm of *B. mojavensis* was found to grow in Berea sandstone cores under 8.9 MPa and 32°C (Mitchell et al., 2008). Under the condition of 50–80°C and 271 atm with supercritical CO_2_, spore-forming organisms, such as *B. subtilis*, *B. cereus*, and *B. pumilus*, showed a 0.58–3 log_10_ reduction in viability over a 4-h period (Zhang et al., 2006). Similarly, *S. pasteurii* were reported to maintain viability at a pressure of 7.5 MPa (Mitchell et al., 2013). Deep-sea microorganisms, such as barophiles, are possible candidates, with their ability to survive under extreme pressure conditions (Abe et al., 1999). Research on mesoscale high-pressure experiments and modeling toward the field-scale implementation is being undertaken (Phillips et al., 2012).

In the field application where hypersaline aquifer is present, microorganisms growing in highly saline environments can be considered. It is reported that *S. pasteurii* can sustain salinities up to 58 g/L (Kuhlmann and Bremer, 2002), and they were able to increase pH and speed up the carbonate precipitation within the range of 5.8–35 g/L (Dupraz et al., 2009b). In the bioengineering projects under high temperature, strains isolated from hot springs (Fouke, 2011; Okumura et al., 2013) can be considered to increase the carbonate precipitation.

#### Diminishing Undesired By-products

To mitigate the undesired by-products, several solutions can be adopted: (1) find alternative strategies to avoid the generation of these secondary products, (2) use the by-products for other applications nearby, and (3) adopt treatments to dismiss the by-products.

To avoid the generation of undesired by-products, alternative techniques based on microbial cells can be adopted for carbonate precipitation. For example, biomineralization takes place on cell walls or EPS serving as a nucleation template, or alternative metabolic activities, such as photosynthesis and denitrification, which do not result in undesired end-products, offer viable options.

In the case of denitrification, toxic intermediate products can be avoided by ensuring the completeness of reactions. The generated by-products can be repurposed in other applications. For example, ammonia released from ureolysis and ammonification can be used in the fertilizer (De Jong et al., 2013).

A third solution is to treat the generated by-products after the application is done. Biological reactors with microbial consortia have been applied to treat wastewater, including ammonia (Grady and Filipe, 2000; Herrero and Stuckey, 2014).

#### Control the Growth of Bacteria

Strategies need to be taken to control the growth of introduced or augmented bacteria. One solution is to control the source by using inactive cells, such as killed or freeze-dried microorganisms. Spore-forming bacteria can survive longer; therefore, they are less preferable in this aspect. Another solution is to limit the availability of nutrients after application and lead to the die-off of bacteria.

### Toward Engineering Technologies

#### Bioreactor

To improve the functionality of the bioreactors, both viability of microorganisms and mechanical performance of the system have to be ensured. Parameters such as temperature, pH, cell density, pressure, and, in some cases, light intensity are essential factors for microbial activities. Higher surface area-to-volume ratio, well mixing, scalability, and ease of operation are crucial features to improve the bioreactor systems (Kumar et al., 2011). In the case of carbon sequestration, SO_x_ and NO_x_, critical CO_2_ concentration and O_2_ accumulation should be considered.

To decrease the cost of the real application, nutrients and cells are not necessarily of the same purity grade as those in the laboratory. For example, corn steep liquor and lactose mother liquor can be economical alternatives of medium ingredients to lower the cost (Achal et al., 2009, 2010). To avoid the high cost due to the use of pure bacterial cultures, approaches with mixed or non-axenic bacterial cultures should be developed (Silva et al., 2015).

#### Injection Designs

By controlling key field parameters such as concentrations of chemicals, flow rates and amendment injection (Lauchnor et al., 2013), and distribution of injection wells with a deliberate pattern (De Jong et al., 2013), an optimal injection strategy can be achieved. Compared with continuous injection, pulsed injection reduced the accumulation of localized mineral plugging, and resulted in a more efficient precipitation of calcium and strontium (Lauchnor et al., 2013). Combined with a controlled flow rate, the pulsed injection can control the distribution of precipitation without extensive plugging of the transport channels. The transportation efficiency can also be improved by designing a pattern of injection wells according to the morphology and property of the remediation site. With further studies on optimizing injection strategies, including single- versus multiple-phase injections, surficial flooding versus deep injection, and high-concentration versus low-concentration treatments (De Jong et al., 2013), it will be possible to develop an ideal injection design for the field-scale applications.

#### Obtaining Uniform Treatment

The original non-uniform formation subsurface structure makes it challenging to obtain a homogeneous treatment with MCP. By modifying injection patterns and changing flow directions, it is possible to improve the efficiency. For example, by adopting an injection pattern with five-spot wells, and injected fluids in opposite directions, a relatively uniform treatment was achieved in the field application (Dejong et al., 2014). In addition, bacteria in nutrient solution were re-circulated resembling a large-scale chemostat, which keeps a uniform distribution of highly active microbes. Other parameters, such as solution viscosity and density, and microbe size relative to soil pore throat size can be managed to optimize the treatment.

#### Monitoring

The monitoring of the MCP in the field application is challenging mostly due to the inaccessibility of sampling sites. Remote sensing and an automatic system can be possible solutions. Soil properties of the remediated site can be obtained through remote sensing, and a map showing spatial variability will be produced. Another approach can be installing an automatic sampling and analyzing system, coupled with a quality-control program.

## Author Contributions

TZ and MD have contributed to the manuscript equally, reviewed the literature and organized the structure of the manuscript, and wrote the manuscript and approved the final version to be published.

## Conflict of Interest Statement

The authors declare that the research was conducted in the absence of any commercial or financial relationships that could be construed as a potential conflict of interest.
